# High numerical aperture multilayer Laue lenses

**DOI:** 10.1038/srep09892

**Published:** 2015-06-01

**Authors:** Andrew J. Morgan, Mauro Prasciolu, Andrzej Andrejczuk, Jacek Krzywinski, Alke Meents, David Pennicard, Heinz Graafsma, Anton Barty, Richard J. Bean, Miriam Barthelmess, Dominik Oberthuer, Oleksandr Yefanov, Andrew Aquila, Henry N. Chapman, Saša Bajt

**Affiliations:** 1 Center for Free-Electron Laser Science, DESY, Notkestrasse 85, 22607 Hamburg, Germany; 2 Photon Science, DESY, Notkestrasse 85, 22607 Hamburg, Germany; 3 Faculty of Physics, University of Bialystok, K. Ciolkowskiego 1L, 15-245, Bialystok, Poland; 4 SLAC National Accelerator Laboratory, 2575 Sand Hill Rd., Menlo Park, CA 94025, USA; 5 Dept. of Physics, University of Hamburg, Luruper Chaussee 149, 22607 Hamburg, Germany; 6 European XFEL GmbH, Albert Einstein Ring 19, 22761 Hamburg, Germany; 7 Centre for Ultrafast Imaging, Luruper Chaussee 149, 22607 Hamburg, Germany

## Abstract

The ever-increasing brightness of synchrotron radiation sources demands improved X-ray optics to utilise their capability for imaging and probing biological cells, nanodevices, and functional matter on the nanometer scale with chemical sensitivity. Here we demonstrate focusing a hard X-ray beam to an 8 nm focus using a volume zone plate (also referred to as a wedged multilayer Laue lens). This lens was constructed using a new deposition technique that enabled the independent control of the angle and thickness of diffracting layers to microradian and nanometer precision, respectively. This ensured that the Bragg condition is satisfied at each point along the lens, leading to a high numerical aperture that is limited only by its extent. We developed a phase-shifting interferometric method based on ptychography to characterise the lens focus. The precision of the fabrication and characterisation demonstrated here provides the path to efficient X-ray optics for imaging at 1 nm resolution.

Hard X-rays are ideal for high-resolution imaging and spectroscopic applications due to their short wavelength, high penetrating power, and chemical sensitivity. Many imaging and micro-spectroscopy applications require a focusing optic to produce a small beam that can be rastered over an object or used to form a magnified image of the object. In these cases the spatial resolution, *δ*, of the image depends on the numerical aperture (NA) of the optic, following the same relationship for optical lenses, *δ* = 0.61 *λ*/NA (for incoherent imaging) where *λ* is the wavelength. The penetrating power that makes X-rays useful for imaging also makes focusing them technologically challenging. For high-energy X-rays the refractive index of suitable lens materials differs from unity by approximately 10^−5^, which means that a large number of refractive surfaces are required to deflect or focus a beam^1^. The state of the art in focusing hard X-rays has been achieved by reflection from a curved mirror at grazing incidence to produce a 7 nm spot size^2^. This required fabricating mirrors of large surface areas (over 8 cm long) with nanometre figure accuracy. Diffractive lenses based on Fresnel zone plates have been successfully used to obtain images with about 10 nm Rayleigh resolution^3^ with soft X-rays. These devices can be described as concentric circular transmission gratings in which the grating period (zone period) decreases with radius to diffract rays to a common axial focal point, a relationship referred to as the zone-plate condition. Again, the properties of materials limit their use at shorter wavelengths. For example, to achieve a π phase shift in alternating zones requires the X-rays to transmit through a thickness of about 10^5^ half wavelengths, or about 5 μm thickness for 1 Å wavelength. At the same time, the focussed spot size matches the outermost zone period, implying an aspect ratio of 500:1 for those zones if a 10 nm spot size is to be achieved. Not only are such structures difficult to fabricate using conventional lithographic methods, but their high-aspect ratio prevents a simple thin-mask description of X-ray diffraction. In particular, such structures are akin to planes in a crystal, in which X-rays only reflect when they are tilted at the Bragg angle *θ* (given by sin *θ* = *λ*/(2 *d*), where *d* is the zone period). This comparison is indeed very apt and provides the insight into constructing an efficient hard X-ray lens of high resolution which ideally consists of reflecting confocal parabolic layers (for an incident plane wave) spaced apart such that each period introduces an additional wavelength of path for the rays arriving at the focus^4^. That is, the lens is composed of layers that simultaneously follow the zone-plate condition and are oriented to obey Bragg’s law across the entire lens aperture. The lens performance is described by dynamical diffraction, and as such the optical thickness of the lens should be set at half a pendellosung period to direct most of the incident beam into the diffracted (focused) beam, giving much higher efficiency than could be achieved with a thin zone plate (which is limited by equally partitioning the beam into positive and negative orders).

A method to fabricate volume zone plates of high aspect ratios was introduced a decade ago^5^,^6^,^7^. Called multilayer Laue lenses (MLLs)^8^, these structures are fabricated by layer deposition, using technologies developed for making multilayer mirrors^9^. Layer periods thinner than 1 nm are achievable by magnetron sputtering^10^. Lenses are made by alternately depositing two (or more) materials with layer periods that follow the Fresnel zone-plate condition and then slicing the structure approximately perpendicular to the layers to the desired optical thickness. Lenses fabricated to date have consisted of parallel layers in a one-dimensional (1D) stack deposited onto a flat substrate. Two-dimensional focusing can be achieved with crossed 1D stacks^6^,^13^ or by depositing a multilayer on a thin wire to create a circular multilayer zone plate^11^,^12^. In the former case each lens must be tilted relative to the incident X-ray beam to maximize the region of the lens that satisfies Bragg’s law. Even so, the NA of the lens will depend on the rocking-curve width of the Laue reflection (which unfortunately becomes narrower as the thickness of the lens and efficiency of the Laue reflection is increased or as the layer period is reduced). A tilted MLL consisting of parallel layers was used to focus 12 keV X-rays to a spot of 11.2 nm (FWHM) with 15% efficiency^14^. When the NA of the lens exceeds the Darwin width of the reflection at any part of the lens then the lens focus will be significantly apodised and the effective NA will be limited by the diffraction efficiency. Only by varying the tilt of the layers throughout the stack, so that Bragg’s law and the zone plate condition are simultaneously fulfilled for every layer, is it possible to construct a large enough NA to focus X-rays to nanometer spots. Such a structure is referred to as a wedged MLL, and is schematically illustrated in Fig. 1 (a).

Numerical modelling of MLLs has been carried out using methods such as coupled wave theory, the beam propagation method (equivalent to the multislice technique), and dynamical diffraction of distorted lattices. In the latter case, it was predicted that efficient wedged MLLs with NAs as high as 0.1 should be achievable; that is, focal spots smaller than 1 nm should be possible using wedged MLLs^15^. Until now, however, a wedged MLL has not been realized experimentally, due to difficulties in controlling material deposition to the necessary precision both in the direction of the film growth and transverse to this direction.

Recently, we solved the manufacturing problem of wedged MLLs by depositing the layer materials by magnetron sputtering onto a substrate shadowed by a straight-edged mask^16^. The required layer period and layer angle was achieved in the penumbra of the mask where the deposition rate changes with distance in a direction perpendicular to the mask edge. Here we present the measured one-dimensional (1D) focusing performance of a high-NA wedged MLL made in this fashion, and compare this performance to calculations based on the beam propagation method (see Methods). We find that the diffraction efficiency is nearly uniform across the entire pupil of the lens, which had a NA of 0.006 at 22 keV photon energy. The depth of the lens (the thickness in the direction of the optical axis) was 6.5 μm, which gives a computed efficiency of 60 ± 1% for a perfect structure. We characterized the focused wavefield by pytchography^17^,^18^,^19^,^20^,^21^,^22^,^23^ using a 95 nm period transmission grating as a test structure. By carrying out ptychographic measurements (far-field diffraction patterns as a function of the transverse position of the object) with the grating placed at various defocus positions (Fig. 2) we recovered the focal properties along with the structure of the grating. We determined a focal spot size of 8.4 nm using the Rayleigh criterion, despite a phase defect in the pupil of the lens.

## Results

### Optimisation of the photon energy

**Figure 1:**
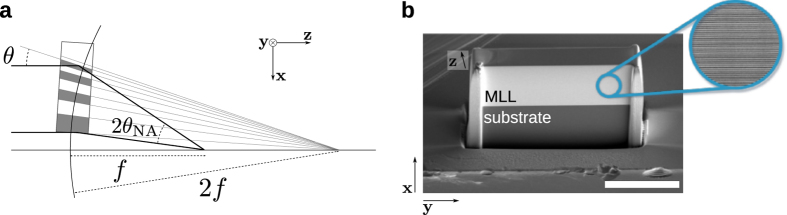
(**a**) A wedged multilayer Laue lens of focal length *f* is constructed from layers whose spacing follows the zone-plate condition. To achieve high efficiency the lens must be thick, in which case diffraction is a volume effect described by dynamical diffraction. In this case the layers should be tilted to locally obey Bragg’s law, which places them normal to a circle of radius 2*f*. (**b**) SEM image of the 2750-bilayer wedged MLL used in this study. The regions corresponding to the multilayered materials and the Si substrate are indicated. The white scale bar is 20 μm and the inset shows a magnified TEM image of the layered materials.

**Figure 2:**
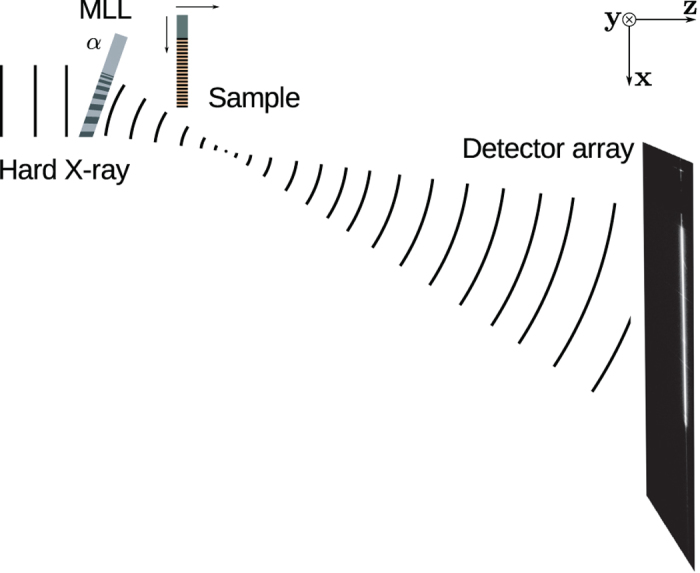
Experimental arrangement of the X-ray measurements, indicating the orientation of the coordinate system and the tilt of the lens, α. Diffraction patterns were recorded on a Lambda detector located 3.4 m from the MLL. Ptychograms were measured from a thick transmission grating of 95 nm period and was scanned in the *x* direction at various defocus distances.

The wedged MLL was designed and prepared to operate at photon energies close to 17 keV with a 1.2 mm focal length and a 5 nm spot size (see Methods). The fabricated MLL, measuring 40 μm wide, 17.5 μm high and 6.5 μm deep is shown in Fig. 1 (b). The variation in the tilt of the layers of the MLL throughout the stack was achieved by slicing the lens from a structure deposited in the penumbra of a straight-edge mask. In order for all layers to obey Bragg’s law they must be tilted so that incident collimated rays reflect from the layers to the focus, which occurs if all layer surfaces are normal to a circle of radius 2*f*. At the same time the spacing of layers in the stack must satisfy the zone plate condition for the focal length *f* in which the grating period decreases inversely with its distance from the optic axis so that waves are diffracted to a common point at *f*. The tilt is a geometric property that does not change with wavelength, whereas the zone-plate focus varies inversely with wavelength. Thus, our fabricated MLL is optimised only for the wavelength where the zone-plate condition matches the curvature condition of the layers, which depends on where in the penumbra the lens is sliced. The optimum wavelength was determined empirically from measurements of the diffraction efficiency across the lens at various wavelengths, which was achieved by measuring the far-field diffraction of the lens (3.4 m from the lens) with a monochromatic and collimated X-ray beam and using a pixelated detector with square pixels of 55 μm width (see Methods). A schematic of the experiment is shown in Fig. 2, which also shows the definition of our orthogonal coordinate system: *z* along the optic axis; *x* transverse to *z* in the direction of the lens focusing; and *y* in the non-focussing direction. Thus, the 40 μm width of our MLL is positioned approximately in the *y* direction, the 17.5 μm height in the *x* direction, and the 6.5 μm depth in the *z* direction, as shown in Fig. 1 (b).

**Figure 3:**
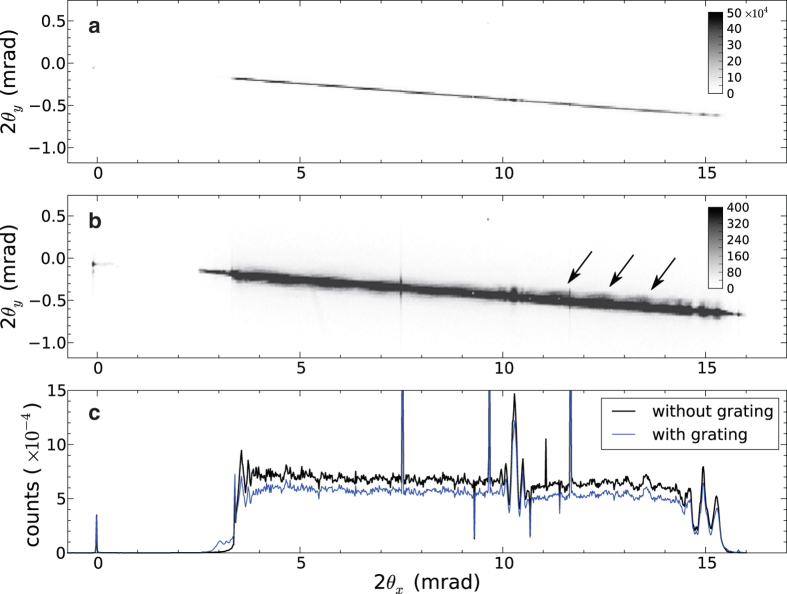
The far-field diffraction of the lens measured without any sample in place (**a**), and with the test grating in the beam 80 μm out of focus (**b**). Also shown in (**c**) are line-outs of intensity formed by integrating over detector columns (in *y*) without (black) and with (blue) the sample in place.

A far-field measurement of the diverging beam from the focus of the MLL is shown in Fig. 3 (a) for a photon energy of 22 keV (0.056 nm wavelength). The beam width in the unfocussed (*y*) direction matches the 40 μm width of the lens and is less than the width of a single pixel. However, the lens was tilted about the optic axis by about 2° relative to the detector rows and so occasionally the beam is split over two pixels. A plot of the intensity formed by summing over detector columns is also shown in Fig. 3 (c), which immediately shows a near-uniform diffraction efficiency across the entire pupil of the lens. The other major feature in this plot is a spike in intensity near a deflection angle of 2*θ* = 10 mrad. As described below, this is due to a localized phase defect in the lens. The absolute diffraction efficiency was not measured (since the incident beam overfilled the MLL).

**Figure 4:**
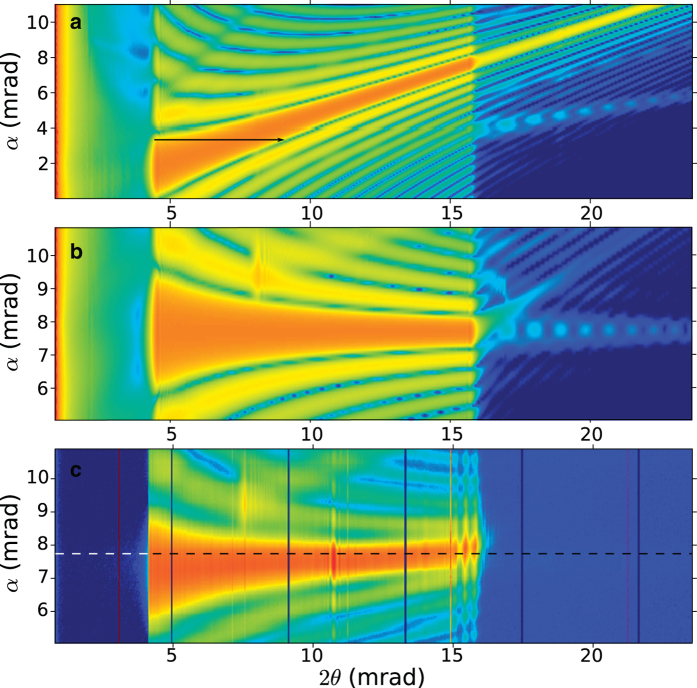
Diffraction efficiency (logarithmic scale), as represented by the far-field 1D diffraction of the lens, as a function of tilt (α) of the MLL lens. Simulations of MLLs with parallel layers (**a**) and with wedged layers **(b**) show that high NA is only achievable with the latter. (**c**) Measurements of diffraction from our wedged MLL. The stage used to tilt the lens was not encoded and so the tilts are only approximate. The arrow indicates the region of the pupil corresponding to the largest NA in (**a**). The dashed line highlights the tilt angle used for subsequent analysis. Note the difference in the range of tilts in (**a**).

We determined the optimum wavelength and geometry by mapping the diffraction efficiency as a function of the tilt of the lens about the *y* axis. Changing this angle α changes the Bragg angles of all layers in the lens. When the wavelength-dependent zone-plate condition is not matched to the layer curvature then the diffraction efficiency will not be satisfied across the entire lens pupil, and by varying α the region of maximum diffraction efficiency will vary across the pupil. When the wavelength is matched to the layer curvature then there will be one angle α that gives optimum diffraction efficiency across the entire pupil. This behaviour is illustrated in Fig. S1 (Methods), where the simulated far-field diffraction is mapped as a function of lens tilt α for four different photon energies, and compared with measurements of our MLL. It is quite clear from the simulations and measurements that 18 keV is not optimum energy since there is no single lens tilt that gives uniform diffraction efficiency across the lens. Instead, as in the case of non-wedged MLLs, the diffraction efficiency reduces the numerical aperture of the lens. We find uniform efficiency across the entire pupil is obtained at 22 keV. This is a shorter wavelength than we anticipated based on the profile of the deposited multilayer structure, but not unexpected given the accuracy in determining the position in the penumbra where the lens was cut. Despite this, it is clear that the wedged profile of the layers is very close to ideal since a photon energy can be found for which the scattering efficiency is nearly uniform across the pupil function. In Fig. 4 we compare the simulated diffraction efficiency for a parallel and wedged MLL, shown in (a) and (b) respectively, with our experimental results in (c) for 22 keV X-rays. This clearly illustrates that the NA of the parallel MLL is limited by the diffraction efficiency across the lens pupil. We also observe good qualitative agreement between the simulated and observed diffraction efficiencies.

We subsequently performed all further measurements at a photon energy of 22 keV and at the optimum tilt angle of α = 7.8 mrad, as indicated by the dashed line in Fig. 4 (c). This is also the condition for the measurements shown in Fig. 3. For this condition the convergence of the focused beam ranges across the entire pupil from a scattering angle 2θ = 3.5 mrad to 15.5 mrad (or 2θ_NA_ = 12 mrad), corresponding to NA = 0.006 at a wavelength of 0.056 nm, which would give a focal spot size no smaller than 0.61λ/NA = 5.7 nm if the lens had no aberrations.

### Focus characterisation by ptychographic interferometry

Knowledge of the pupil aberrations of a lens completely characterises its focal properties, since the complex-valued pupil function is related to the focal distribution by a Fourier transform, and indeed a coherent wavefield measured at one plane can be numerically propagated to any other plane. Aberrations in zone plates are primarily caused by placement errors of the zones. A misplacement of a zone by one tenth of a period, for example, will cause a π/5 phase error. For the outermost zones which have a 3.7 nm period, the layers would need to be placed to an accuracy better than 0.37 nm in order not to cause significant aberrations. While this sounds technologically challenging, low order variations in deposition rate may lead to errors that can be simply compensated by focus adjustment.

There is a rich history of wavefront characterisation of optical elements^18^, including interferometric and non-interferometric methods such as the Foucault (or knife-edge) test. Ptychography^17^ can be thought of a generalisation of many of these methods^19^ and involves recording the far-field diffraction from the convergent beam transmitting through a diffracting test object for various transverse positions of that object relative to the beam. A set of diffraction patterns as a function of the scan position of the object is referred to as a ptychogram. In the case of a grating test object, as we use here, the ptychogram can be thought of as a set of phase-shifted shearing interferograms of the lens pupil. Although knowledge of the test object and the (sufficiently sampled) ptychogram completely specifies the amplitude and phase of the incident wavefield (the probe), phase retrieval methods have been adopted to simultaneously recover the complex-valued probe wavefield and the complex-valued transmission function of the object^17^,^20^. This is possible due to the large degree of redundancy in the measurements when the displacements of the object in the scan are smaller than the beam spot at the test object, giving rise to regions of overlap on the test object that are measured with high diversity. Furthermore, redundancy in the data is improved when the illuminated area of the test object is increased, allowing an increase of the overlap length between adjacent scan positions. Thus defocusing the sample helps, rather than hinders, the characterization process. Due to its robustness and ability to recover both the object and beam amplitude and phase maps, ptychography is a favoured way to characterise X-ray optical elements such as zone plates and MLLs^13^,^21^,^22^,^23^.

A single far-field diffraction pattern with a grating test object located 80 μm out of focus is shown in Fig. 3 (b), along with the projected lineout in Fig. 3 (c) (shown in blue). The transmission grating consisted of 20 bilayers with 95 nm period and a thickness along the beam of 4 μm (see Methods). Compared with the diffraction pattern without any test object, Fig. 3 (b) shows weak oscillations due to the interference of the zero and first order diffraction of the grating. The zero-order beam is essentially the same as that without the grating as shown in Fig. 3 (a) and the first-order beam consists of another copy of this shifted by the diffraction angle of the grating: 2*θ*_p_ = λ/*d* = 0.6 mrad, where *d* = 95 nm is the grating period. Since the grating was out of focus by Δ*z* = 80 μm, the diffracted beams carry wave curvature (or a quadratic phase variation) which in the shearing interferogram gives an oscillation of period equal to
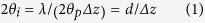
and can be thought of as a projection image of the grating. (Another way to envision this is that the grating causes a shifted copy of the focus separated by 2*θ*_p_ Δ*z* giving rise to two interfering waves.)

**Figure 5:**
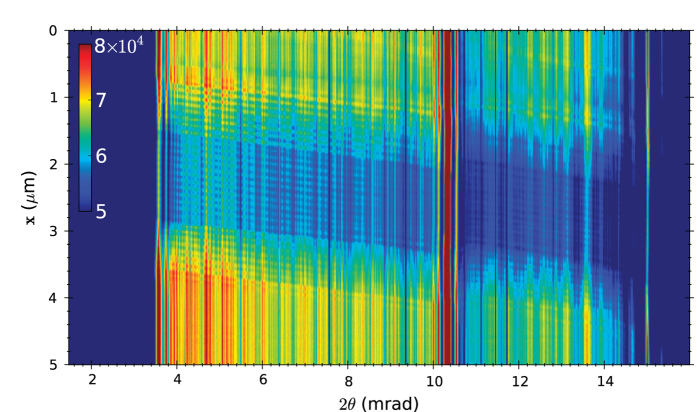
A 2D ptychogram of the defocussed grating (Δ*z* = 80 μm) formed from projections of diffraction patterns measured as a function of the grating position, *x*. Each value in this map, represented by the color scale, is the total number of scattered photons for a particular scattering angle 2*θ* and grating position *x*.

**Figure 6:**
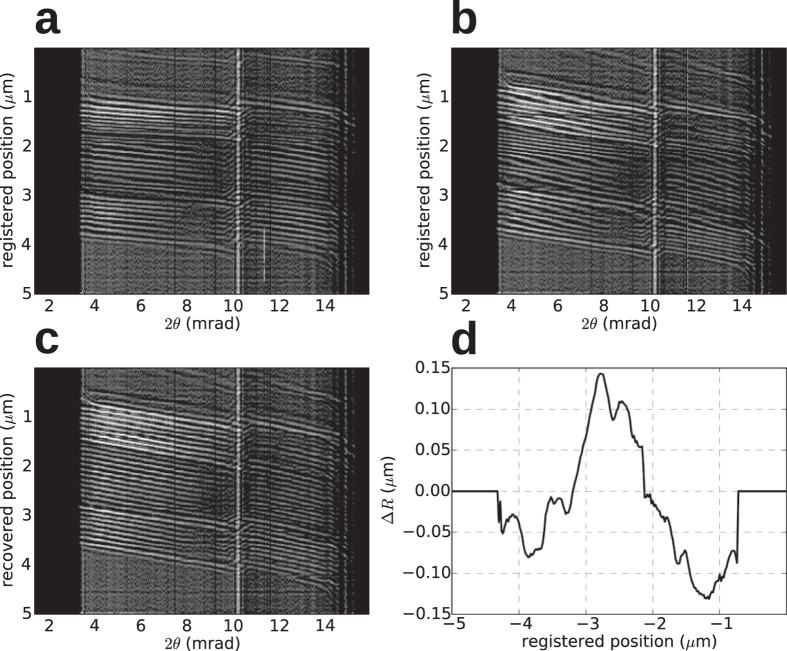
A fringe analysis of the defocussed ptychograms can be used to determine stage positioning errors. Fourier filtered ptychograms, highlighting the fringes caused by interference of grating orders, for a grating defocus of (**a**) 30 μm and (**b**) 80 μm (**c**) Filtered ptychogram after correction of the stage errors (**c**). (**d**) Plot of the residual stage errors, with a standard deviation of 65 nm.

A 2D ptychogram with the grating is shown in Fig. 5. Each row of the pytchograph is a diffraction lineout such as those shown in Fig. 3, for each position *x* in the scan of the grating object made in Δ*x* = 20 nm steps. One striking feature of the map is the set of tilted fringes. The tilt is due to the shifting of the interference fringes with the stepping of the grating. As the grating is brought closer to the focus the fringes become broader with scattering angle 2*θ* as noted by equation (1). Any column of the ptychogram is a scanned coherent image of the grating^21^,^22^,^23^. Stepping an infinite grating through a full period returns to the same pattern, and hence coherent images (columns) have periods equal to the 95 nm grating period. Thus, if the grating is placed closer to the focus, the fringes in the 2D ptychogram will become less tilted, becoming horizontal at best focus, and reversing direction on the other side of focus. Indeed, from equation (1) the tilt of the fringes in the ptychogram are given by *d /* (2*θ*_i_) = Δ*z* and hence this tilt gives a convenient, accurate, and direct read out of the defocus. This point is emphasised in a fringe analysis of two ptychograms, displayed in Figs. 6 (a) for defocus values of Δ*z* = 30 μm and 80 μm, respectively. The tilt can also be thought of in terms of the magnification of the projection image of the grating formed by the focus. As the defocus is increased the magnification is reduced and the field of view of the object illuminated by the angular extent of the beam (equal to 2 NA Δ*z*) is increased. The incoherent scanned image, formed by projecting the ptychogram onto the *x* axis, should resolve the grating features when the grating is within the depth of focus where the fringe tilt is less than a grating period across the angular span of the lens (unlike the out-of-focus scanned image which integrates along 2*θ* over the tilted fringes, reducing the contrast).

A second feature of the ptychogram in Fig. 5 is that there are more periods in the *x* direction than the 20 periods of the grating. This is due to the fact that the thick grating was tilted about the *y* axis by about 10°. The 95 nm period grating was fabricated with a 4 μm thickness along the *z* direction to provide a significant diffraction efficiency for the wavelengths used here, and was tilted to achieve predominantly just a zero and first order diffracted beam. However, at such a tilt the back of the grating was misregistered from the front by more than 700 nm, or about 7 periods. Instead of the transmission through 20 rectangular-shaped periods we thus expect to observe an extra 7 periods, with a transmission of the first and last 7 periods varying with distance from the edge of the grating structure. This gives rise to the variation in intensity observed in the *x* direction of the ptychogram.

Yet a third prominent feature of Fig. 5 is the collection of vertical lines, especially the strong intensity at a scattering angle of 2*θ* = 10.3 mrad. Looking more closely, it is seen that the tilts of the fringes on either side of this discontinuity differ, and the fringes are bent at a scattering angle just above the discontinuity. This feature corresponds to a phase error in the pupil, and it is clear from the fringe tilts that the two regions of the lens correspond to two modes each with a different focal plane, separated by Δ*z* = 17.2 μm. We speculate on the origin of this error below. The other vertical lines in the ptychogram are due to variations in diffraction efficiency in the lens, which are not as high contrast as they appear due to the colour scale of the map (chosen to maximise the contrast of the interference fringes).

Aberrations are visualised by any non-straightness of the fringes: a displacement of a fringe in the 2*θ* direction is proportional to the phase gradient. Within each of the regions of the lens the fringes are mostly straight, indicating low aberrations. However, the fringes can also be crooked or distorted due to positioning errors in the stage that moves the grating. We can distinguish the effect of stage errors from lens aberrations in the defocused ptychograms because a localised error in the stage position will give rise to a distortion at a particular *x* of all fringes in the ptychogram across the full range of 2*θ*, whereas a localised phase error will give rise to a distortion localised in 2*θ* (dramatically illustrated with our MLL). Distinguishing these sources of error does not require accurate knowledge about the test object, nor does it require a strictly periodic object. Again, it is the redundancy in the data that achieves this; with a large defocus we illuminate a large number of grating periods in a single exposure, and there are many exposures that include a particular feature of the object. The errors can be addressed through a fringe analysis of defocussed ptychograms, such as shown in Figs. 6 (a) for two defocus distances (see Methods). In particular some distortions of the fringes at localised *x* values (i.e. horizontally) are indeed visible, which are improved in the corrected ptychogram shown in Fig. 6 (c) for comparison with (b). The difference between the nominal and retrieved stage positions are shown in Fig. 6 (d). The standard deviation of the corrections was 65 nm.

### Ptychographic reconstruction of the focus

After correction of the ptychograms to account for the stage errors we carried out iterative phase retrieval of the probe wavefield and test object transmission independently for various defocus positions of the grating (see Methods). This allowed us to cross check the results from each defocus position, providing confidence in the recovered beam profile.

**Figure 7:**
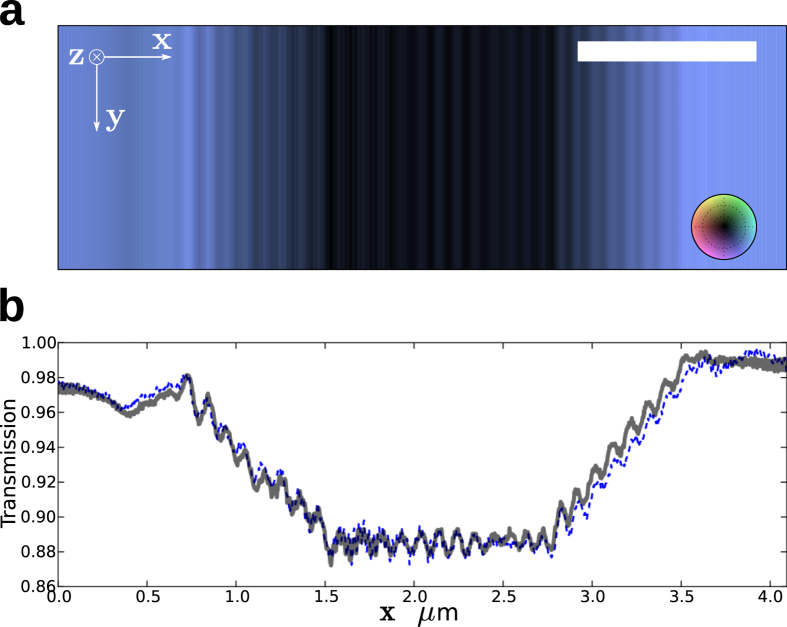
(**a**) The retrieved complex transmission of the grating. The brightness of the image is proportional to the amplitude of the transmission, while the hue indicates the phase (as shown by the color wheel). The white scale bar indicates 1 μm (**b**) Lineouts of the amplitude of the grating transmission retrieved independently from ptychograms recorded at Δ*z* = 70 μm (blue) and 80 μm (grey).

Figure 7 (a) shows the reconstructed complex-valued transmission of the test grating. The brightness of this image is proportional to the amplitude of the transmission, and the hue to the phase. The amplitude is also plotted in Fig. 7 (b) with the blue and grey lines corresponding to independent reconstructions from ptychograms measured at 70 μm and 80 μm defocus distances respectively. The two reconstructions are in close agreement. As expected from the interferometric analysis, the fact that layers of the thick grating were not parallel to the beam propagation direction caused a significant drop in the contrast of the grating transmission. The tilt gave rise to two regions where the transmission (averaged over a period) varies linearly and a third central region of 12 periods with uniform average transmission where the front of the grating layers overlap with the rear.

**Figure 8:**
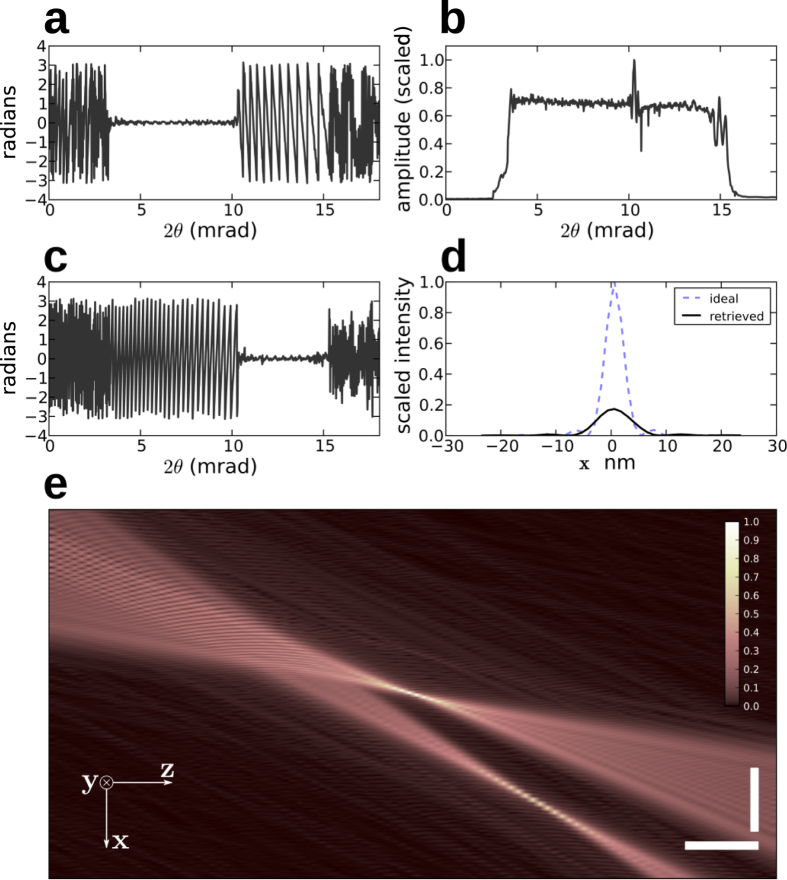
Line profiles of the phase (**a**) and amplitude (**b**) of the retrieved pupil function of the MLL. The phase error in the region of the lens for 2*θ* > 10 mrad is primarily defocus; (**c**) the phase profile of the MLL after propagating by 17 μm in *z* and shifting the optical axis by 0.34 μm in *x*. (**d**) The recovered spot intensity profile (solid line) compared with the ideal intensity profile that would be achieved with zero phase aberration (dashed line). The field near the beam waist is related to the pupil function by a Fourier transform, and is shown (**e**) in a sagittal plane by numerical propagation along *z*. The vertical and horizontal scale bars are 200 nm and 10 μm respectively.

The recovered phase and amplitude of the lens pupil are shown in Figs. 8 (a). The two regions of the lens, separated by the intensity spike observed in the far-field diffraction pattern, clearly reveal a difference in focus. The pupil phase is also shown in Fig. 8 (c) after propagation by 17 μm in *z* and shifting the optical axis by 0.34 μm in *x*, where the high-angle region of the lens is in focus. It is seen that individually each of the lens regions has very low aberration. The RMS phase error in the low-angle region is 0.27 rad (0.04 waves), whereas the high-angle region (excluding defocus) has 0.47 rad (0.07 waves) RMS phase error. Such low errors are unprecedented in hard X-ray optics, for example 0.1 waves in^2^. The reconstructed probe intensity (square of the amplitude) is plotted in Fig. 8 (d) at the plane of best focus for the low-angle portion of the lens. The focal spot as determined using the Rayleigh criterion is 8.4 nm. On the same graph we plot the intensity that would be achieved with zero phase error in the lens and with the same retrieved amplitude, giving a spot size of 4.8 nm. A map of the amplitude of the beam in a sagittal plane is shown in Fig. 8 (e), over a region near the beam waist. This was computed by numerically propagating the wavefield. We clearly see the two distinct modes formed by the two regions of the MLL, which bring the beam into focus in different places.

As a final check of the accuracy of the reconstruction, the grating transmission and probe functions retrieved from the ptychogram measured at Δ*z* = 80 μm were used to calculate a ptychogram at Δ*z* = 70 μm for comparison with the ptychogram that was measured at that plane. We find a relative error of *R*-free = 0.16 (see Methods for details), which gives strong confidence in the reconstruction.

## Discussion

Our wedged MLL was fabricated by a very simple and direct method of depositing alternating layers of materials in the penumbra of an edge mask, and the characterisation we have carried out here shows that this method has the necessary precision, at the ångström level, to produce X-ray lenses of high NA that can focus to spot sizes below 10 nm. It is often said that you can’t make what you can’t measure, and such precision requires a robust and highly accurate characterisation technique. We find that ptychography does indeed meet this need, and we demonstrated that it generalises many optical testing methods such as phase-shifting shearing interferometry, and produces verifiable results. By this method we determined an RMS phase error of 0.27 rad (0.04 waves at 0.057 nm wavelength) in the low-angle region of the lens, corresponding to a layer placement precision of 0.23 nm for the thinnest layers. Our testing revealed an unexpected phase error that separates two regions of the lens. This error is disconcerting and will need to be eliminated in order to produce smaller spot sizes. We carried out a detailed examination of the structure of the lens in this region by high-resolution TEM yet could not find any evidence of a defect in the lens or error in the deposition process (such as a missing layer). Our retrieved pupil phase gives some hint to the origin of this error, which is consistent with one part of the lens being deposited with a slightly different length scaling than the other. As to what could cause such an abrupt change in scale, we speculate that this could be due to a phase transition in the microstructure of one of the layers (W) that occurs at a layer thickness of about 3 nm, similar to previous observations^24^. Since the scale error would cause only a change in layer thickness of 0.01 nm, it is no surprise that it was not discernable in TEM images, and it also points to the high level of accuracy possessed by the rest of the fabricated structure.

The uniform diffraction efficiency that we measured across the pupil (Fig. 3 (a)), over an angular range of 12 mrad (NA of 0.006) at a wavelength of 0.056 nm, is in stark contrast to what would be achievable with a MLL fabricated with parallel layers. In that case we predict that the highest NA would be about 0.0024 (calculated from the maximum width of the main diffraction peak), with a pupil apodised by the diffraction efficiency, as indicated by the arrow in Fig. 4 (a). Thinning the parallel-layer MLL would slightly increase its NA, at the expense of efficiency. While we did not directly measure the efficiency of our wedged MLL, other measurements in our laboratory have indicated that our sliced Laue multilayers perform at close to the theoretical performance, which would be 60 ± 1% for this lens. For this first test of our fabrication method we chose modest lens parameters, such as 2,750 layers over a 17.5 μm lens height, and 1.2 mm focal length, which took over 36 hours to deposit^16^. We have previously made multilayer stacks thicker than 70 μm, and foresee the ability to fabricate lenses with five to 10 times higher NA, either by increasing the number of layers and/or by reducing the focal length, either working with a range of layer thicknesses that avoids the phase transition (if that is indeed the cause of the phase error) or with different materials.

By combining two 1D MLLs in a crossed geometry, the beam may be focused in two dimensions^13^. This enables imaging modes such as spectroscopy, fluorescence, absorption and differential phase contrast, tomography and so on at resolutions below 10 nm. However, we note that ptychographic imaging is an extremely powerful and robust imaging technique, as demonstrated here (and elsewhere), which has the ability to create images of higher resolution than given by the spot size of the lens (by measuring and phasing high-angle convergent-beam diffraction patterns). In fact a small spot size is not required, or even desirable. Instead it is the large convergence angle, given by the numerical aperture of the lens that provides robust ptychographic data collection at high resolution. For example, if the NA exceeds the angular extent of the neighbouring Bragg peaks of, say, a protein nanocrystal, then this will cause interference between Bragg reflections in the diffraction pattern. This interference carries the phase information of those Bragg reflections, which in turn could be used to solve for the protein structure.

## Methods

### Wedged MLL fabrication and sample preparation

Building on previous work of manufacturing multilayer structures of large thickness^25^ by magnetron sputtering, we fabricated our lens by depositing alternating layers of SiC and W onto a flat substrate that was shadowed by a straight-edged mask^16^. The required variation of layer period and layer angle was achieved in the penumbra of the mask, where the deposition rate changes with distance in a direction perpendicular to the mask edge. The change in deposition rate must be tailored to the focal length, since in the lens the layers must lie normal to a sphere of radius 2*f*, where *f* is the focal length. This was achieved by setting the mask to substrate distance such that the width of the penumbra is slightly longer than 2*f*, since the layer thicknesses should converge to zero at a distance 2*f* from the position of the lens. The mask to substrate distance was then precisely calibrated by measuring the thickness profile of a deposited test multilayer with a profilometer, while the deposition rate in the unshadowed region was calibrated by measuring the period of deposited test structures by X-ray diffraction. The change in deposition rate with consumption of the sputtering targets was also calibrated. The lens must be sliced at the lateral position *z* along this profile where the gradient and layer thicknesses simultaneously satisfy the Bragg condition and the zone-plate condition for the desired photon energy. As previously noted^16^, for a given multilayer film thickness *h*, we require a transverse gradient of *h*/(2*f*), or a change in gradient of 1/(2*f*) with the change in film thickness. At various *z* positions in the penumbra the layer heights follow the zone-plate condition scaled by a factor proportional to the overall film thickness. A scaling of a zone-plate by a factor *t* multiplies the focal length by a factor *t*^2^ for a given wavelength, whereas the radius of curvature of the layer tilts scales by *t*. Thus, for a given wavelength the multilayer must be cut at the right *z* location that matches both the correct curvature and height. A small error in either the gradient or scale leads to a MLL that is optimized for a different wavelength.

We designed our 1D wedged MLL to give a focal spot size of 5 nm and a focal length of 1.2 mm at a photon energy of 17 keV. Only an off-axis portion of a full zone-plate was chosen, consisting of 2,750 bilayers varying in period from 15.9 nm to 3.7 nm. The lens was deposited starting from the thinnest period to the thickest, to minimize the effect of the accumulation of thickness errors. The largest period was designed to be located 5 μm from the optic axis and the total lens height was 17.5 μm. To match the 1.2 mm focal length a change in lateral gradient with thickness of 1/(2*f*) = 0.042%/μm was required. The lens was cut from the multilayer structure using a focused ion beam (FIB) at the required position determined from the profilometer measurement. It was then thinned to a depth of 6.5 μm. The final MLL, measuring 40 μm wide, 17.5 μm high and 6.5 μm deep, is shown in Fig. 1 (b).

The test sample, used for characterizing the focused X-ray beam, was a grating with a period of 95 nm that was also fabricated by magnetron sputtering. It consisted of 20 equal thickness SiC and W bilayers. Unlike the MLL this multilayer was periodic and had an overall height of 1.9 μm. The sample was cut by FIB in a similar fashion to the MLL and thinned to a depth of 4 μm.

### MLL Simulations

We simulated the performance of the MLL using the beam propagation method. This mature method is used in calculations of electromagnetic waves in different devices such as optical waveguides^26^, and has been used recently to simulate MLL performance^27^. Our approach here is based on the paraxial approximation of the Helmholtz equation (see e.g.^26^). In the case of X-ray radiation, where the refractive index of the media is close to unity, this takes a form which is identical to the time dependent 2D Schrödinger equation, where the time variable is replaced by propagation distance *z*^28^. There are many powerful methods to solve the time dependent Schrödinger equation. Here, we apply an efficient split operator method^29^ that utilises a fast Fourier transform. The simulation is performed in the following steps: first, the incident plane wavefront is propagated along the *z* direction through the pinhole to the entrance of the MLL using Fourier optics^26^. Propagation in the inhomogeneous media of the MLL is then done using the split operator method. The MLL is modelled as alternating regions with complex dielectric constants of W and SiC for the particular photon energy. The boundaries of bilayers of these materials are given by^30^:
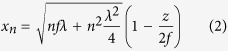
where the curvature 1/(2*f*) causes the change of bilayer thickness (*x*_*n*+1_ − *x*_*n*_) along the propagation (*z*) direction. The wavefront exiting the MLL is propagated in free space to close to the focus, where the intensity is investigated with a fine *z* scan. Finally, the wavefront is propagated to the detector position where the theoretical intensity in the far field is obtained.

### Experimental Methods

X-ray diffraction measurements were carried out at the P11 undulator beamline of the PETRA III synchrotron radiation facility at DESY. The schematic view of our experimental setup is show in Fig. 2. The X-ray beam generated by the undulator was monochromatized with a double crystal monochromator and further collimated and apertured with a 50 μm diameter circular pinhole in 100-μm thick Pt that was placed in line with the MLL. The photon energy was adjustable between 17 keV and 25 keV by tuning the undulator gap and monochromator.

The MLL was held and positioned using an in-house developed translation and rotation stage with three orthogonal translation axes and two rotations about the *x* and *y* axes. The reproducibility of the positioning was better than 5 nm and 5 μrad. The beam diverging from the focus of the MLL was measured in the far field (3.4 m from the focus) using a pixelated X-ray detector. This DESY-developed “Lambda” detector consists of 1536 × 512 pixels formed in a 6 × 2 array of tiles of square pixels, each of 55 μm width^31^. The 95 nm grating was held on a second stage, which also had three orthogonal translations and the ability to rotate the sample only about the *z* axis. The grating was positioned with the variation in its transmission in the *x* direction, such that the grating diffraction was in the focusing direction. A thick (1.1 cm) silicon slab placed just in front of the detector was used to attenuate the unfocussed beam and covered the leftmost 206 detector columns.

Ptychograms of the grating were acquired for a given lens-to-grating distance by measuring a diffraction pattern on the Lambda detector for each position of a stepwise scan of the grating in the *x* direction. Each ptychogram was recorded from 251 sample locations in steps of 20 nm, with an exposure time of 0.5 s per scan position. Between 4 × 10^7^ and 5 × 10^7^ photons were collected in each diffraction pattern.

### Fringe analysis

2D pytchograms of the defocussed grating exhibit fringes that are tilted by an amount that is proportional to the defocus distance. Such defocused pytchograms were Fourier filtered to reveal the fringes, as shown in Fig. 6 (a). The fringes were then indexed by counting both maxima and minima in individual columns and ensuring continuity from column to column. An isolated error in the stage location *x*_*i*_ will distort all fringes by the same amount in that row, which can be distinguished from features in the sample (which follow the tilted fringes) or localised errors in the pupil phase (which distort fringes equally at a particular 2*θ* column of the ptychogram). The error in each position *x*_*i*_ of the stage in the scan was determined by fitting a set of equally-spaced parallel straight lines to the fringes (but allowing for the difference in the fringe tilt in the two regions of the lens separated by the phase discontinuity). The difference between the nominal and retrieved values for *x*_*i*_ are shown in Fig. 6 (d). For the rows in the ptychogram where more than one fringe could not be indexed the values of *x*_*i*_ where not refined (in this case values of *x*_i_ are unconstrained in the phase retrieval, described below).

As a note, we initially attempted to correct for stage position errors using a conjugate gradients algorithm as part of an iterative ptychographic reconstruction, but this was unsuccessful. Additionally, we found that the correction dramatically improved the phase retrieval (discussed below), which converged in 50 iterations compared with many hundreds of iterations without correction.

### Reconstruction algorithm

The pixels at the edges of the 256 × 256 pixel detector tiles of the Lambda detector registered approximately twice the number of photon counts of their neighbours. Rather than correct for the gain of these pixels, they were masked so that their values were allowed to float during the reconstruction process, as were the pixels behind the silicon attenuator.

Although we show 2D ptychograms in Figs. 5,6, formed by integrating each 2D diffraction pattern in the vertical (*y*) direction, we performed reconstructions using 3D ptychograms composed of the full measured 2D diffraction patterns. This produced better results than reconstructing 1D sample and probe functions from the 2D ptychograms, which make the assumption that both the probe and grating were invariant in the *y* direction. This was necessary because in our measurements there was a slight misalignment between the 1D line focus and the grating lines (or the pupil function and the diffraction orders of the grating) that affected the diffraction data. However, since the sample was scanned only in one dimension (*x*), it is possible for erroneous modes to occur in 2D reconstructions of the sample and the probe. This is a special case of the well-known raster-grid pathology in ptychography^20^ where a modulation of the correct transmission map by any function that varies in *y* can also produce a valid solution with a suitable modification of the probe function. We overcame this pathology by constraining the transmission of the sample, but not the probe, to be invariant in *y*. In our final result we revert to 1D solutions by projecting the probe function in the *y* direction.

The reconstruction method seeks to determine the phases for the set of 251 diffraction patterns 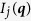 such that each is related (by a Fourier transform) to an exit wave 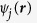
that can be described as an object transmission 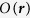 modulated by a shifted probe 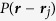, where 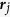 is the known probe displacement for the *j*th diffraction pattern. Here 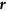 and 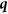 are the 2D transverse components of the real and reciprocal space vectors, respectively. Neither *O* nor *P* are initially known. The phasing is achieved in an iterative fashion where the exit waves are each constrained to be consistent with the measured diffraction patterns, and together constrained to be consistent with a single object function illuminated in various positions 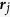 by a single probe function. A particular set of exit waves *ψ* can be represented as a point in a finite-dimensional vector space. Within this formalism, a projection operator maps *ψ* onto the closest point in a constraint set (consisting of those *ψ*_*j*_ that satisfy a particular constraint condition). In particular we utilise P_*F*_ a projection operator that sets the Fourier moduli of *ψ*_*j*_ equal to the square root of the corresponding measured diffraction intensities, and P_*O*_ a projection operator that replaces *ψ* with overlapping *O* and *P* functions that are the closest fit to *ψ*. The simplest iterative phasing algorithm, called error reduction, involves sequentially enforcing each constraint and repeating, such that after the *n*th iteration we have
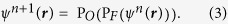
We employed this, alternating with the difference map algorithm (as formulated in^32^) where
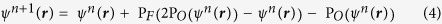


The initial estimate (*n* = 0) was obtained by constructing exit waves from an illumination function derived from the square-root of the measured no-sample diffraction intensity and setting the phase to zero (before adding the estimated defocus aberration from the fringe analysis), modulating a sample transmission initialised with random numbers evenly sampled from a unit circle in the complex plane.

The modulus constraint operator 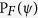 was applied by enforcing the measured diffraction amplitude to each exit surface wave independently
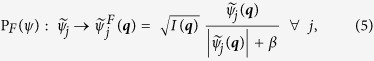
where 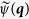 are the Fourier transforms of the real space exit surface waves and *β* = 10^–10^ regularises the division by 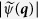. This substitution of diffraction amplitudes is only performed on 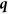 values corresponding to unmasked pixels on the detector; the masked pixels are not updated.

The overlap projection operator P_*O*_ enforces consistency of the exit waves with a single sample function that is modulated with various shifted positions of a single probe function. The operator provides new estimates for the sample and probe functions, 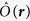 and 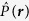, given by a least squares fit to the previous estimate of the set of exit surface waves:
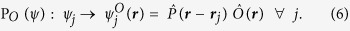
An updated object function 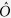 can be obtained from the set of exit waves given the current estimate of the probe function, or *vice versa* for 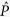. In particular, the updated object function is given by
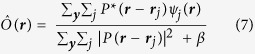


This enforces the 1D constraint placed on the sample by integrating both the denominator and numerator over the constrained dimension (*y*) so that each row (constant *y*) of an exit surface wave is treated as a separate view of the sample. When employing equation (7), and setting 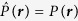 in equation (6), the overlap operator acts by updating only the object function. On the other hand the probe function can be updated by
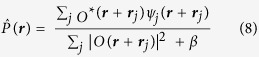
With this, and setting 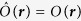 in equation (6) the overlap operator only updates the probe function. A third option is to update *O* and *P* together and iterate between equations (7) and (8) as a way to approximate solving for both choices simultaneously. We find that alternating five times between equations (7) and (8) gives a close approximation to solving these equations simultaneously.

Phase retrieval was carried out in 50 iterations in total, as described in Table 1. The first 25 iterations used the difference map and the last 25 used error reduction. Initially the overlap projection was set to update only the object transmission, using the initial estimate of the probe, and then the projection operator was changed to update only the probe. In the final 25 iterations both the sample and probe functions were updated together. The resulting probe function and sample transmission map were then used to form a forward estimate of the 251 measured diffraction patterns. Borrowing from crystallography we quote the *R* factor for the reconstruction:
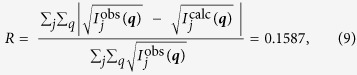
where 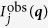 are the recorded diffraction patterns and 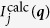 are calculated from the retrieved sample transmission map and probe functions, assuming the relation in Eq. (6). Another error metric borrowed from crystallography is *R*-free; to calculate *R*-free we used the retrieved sample transmission and probe maps obtained from the sample grating at Δ*z* = 80 μm to forward simulate and compare with the diffraction data independently measured with the sample at Δ*z* = 70 μm. To do this we numerically propagated the retrieved probe 10 μm in –z to simulate the incident probe in the new plane. We then accounted for an overall shift in the sample to probe displacement vectors by calculating the *R*-free for a range of global displacement offsets, choosing the smallest value. We obtained an *R*-free of 0.1601. This indicates that the probe and sample transmission map are not under constrained in the ptychographic retrieval.

## Additional Information

**How to cite this article**: Morgan, A. J. *et al*. High numerical aperture multilayer Laue lenses. *Sci. Rep.* **5**, 9892; doi: 10.1038/srep09892 (2015).

## Supplementary Material

Supporting Information

## Figures and Tables

**Table 1:** The sequence of algorithm and overlap update projection used in the ptychographic iterations to reconstruct the sample transmission map and probe functions.

***n***	**algorithm**	**update**
1 → 5	difference map	transmission map
6 → 25	difference map	probe
26 → 50	error reduction	transmission map / probe

Values for the iteration index (*n*) in the first column are inclusive.

## References

[b1] Snigirev A., Kohn V., Snigireva I. & Lengeler B. A compound refractive lens for focusing high-energy X-rays. Nature 384, 49–51 (1996). 10.1364/ao.37.000653 18268637

[b2] Mimura H. *et al.* Breaking the 10 nm barrier in hard-x-ray focusing. Nat. Phys. 6, 122–125 (2010).

[b3] Chao W., Fischer P., Tyliszczak T., Rekawa S., Anderson E. & Naulleau P. Real space soft x-ray imaging at 10 nm spatial resolution. Opt. Express 20, 9777–9783 (2012). 22535070 10.1364/OE.20.009777

[b4] Yan H. *et al.*. Takagi-Taupin description of x-ray dynamical diffraction from diffractive optics with large numerical aperture. Phys. Rev. B. 76, 115438 (2007).

[b5] Kang H. C. *et al.*. Synchrotron x-ray study of multilayers in Laue geometry. Proc. SPIE 5537, 127–132 (2004).

[b6] Maser J. *et al.*. Multilayer Laue lenses as high-resolution x-ray optics. Proc. SPIE 5539, 185–194 (2004).

[b7] Kang H. C. *et al.*. High-efficiency diffractive x-ray optics from sectioned multilayers. Appl. Phys. Lett. 86, 151109 (2005).

[b8] Yan H., Conley R., Bouet N. & Chu Y. S. Hard x-ray nanofocusing by multilayer Laue lenses. J. Phy. D: Appl. Phys. 47, 263001 (2014).

[b9] Spiller E. Low-Loss Reflection Coatings Using Absorbing Materials. Appl. Phys. Lett. 20, 365–367 (1972).

[b10] Windt D. L. *et al.*. W-SiC x-ray multilayers optimized for use above 100 keV. Appl. Opt. 42, 2415–2421 (2003). 12737477 10.1364/ao.42.002415

[b11] Koyama T. *et al.*. Circular multilayer zone plate for high-energy x-ray nano-imaging. Rev. Sci. Instr. 83, 013705 (2012). 10.1063/1.3676165 22299960

[b12] Döring E. *et al.*. Sub-5nm hard x-ray point focusing by a combined Kirkpatrick-Baez mirror and multilayer zone plate. Opt. Express 21, 19311–19323 (2013). 23938848 10.1364/OE.21.019311

[b13] Nazaretski E. *et al.*. Perfomance and characterization of the prototype nm-scale spatial resolution scanning multilayer Laue lenses microscope. Rev. Sci. Instr. 84, 033701 (2013). 10.1063/1.4774387 23556821

[b14] Huang X. *et al.*. 11nm hard X-ray focus from a large-aperture multilayer Laue lens. Sci. Rep . 3, 3562 (2013). 24356395 10.1038/srep03562 PMC3868962

[b15] Yan H. *et al.*. Characterization of a multilayer Laue lens with imperfections. NIMA 582, 126–128 (2007).

[b16] Prasciolu M., Leontowich A. F. G., Krzywinski J., Andrejczuk A., Chapman H. N. & Bajt S. Fabrication of wedged multilayer Laue lenses. Opt. Mater. Express 5, 748–755 (2015).

[b17] Rodenburg J. M., Hurst A. C. & Cullis A. G. Transmission microscopy without lenses for objects of unlimited size. Ultramicrosc . 107, 227–231 (2007). 10.1016/j.ultramic.2006.07.007 16959428

[b18] Malacara D. Optical Shop Testing , 2^nd^ Ed. (Wiley, 1992).

[b19] Guizar-Sicairos M. *et al.*. Phase tomography from x-ray coherent diffractive imaging projections. Opt. Express 18, 18374 (2010). 22108985 10.1364/OE.19.021345

[b20] Thibault P., Dierolf M., Bunk O., Menzel A. & Pfeiffer F. Probe retrieval in ptychographic coherent diffractive imaging, Ultramicrosc . 109, 338–343 (2009). 10.1016/j.ultramic.2008.12.011 19201540

[b21] Rodenburg J. M., & Bates. R. H. T. The Theory of Super-Resolution Electron Microscopy Via Wigner-Distribution Deconvolution. Phil. Trans. Roy. Soc. London A 339, 521–553 (1992).

[b22] McCallum B. C. & Rodenburg J. M. Simultaneous reconstruction of object and aperture functions from multiple far-field intensity measurements. J. Opt. Soc. Am. A 10, 231–239 (1993).

[b23] Chapman H. N. Phase-retrieval X-ray microscopy by Wigner-distribution deconvolution. Ultramicrosc . 66, 153–172 (1996).

[b24] Bajt S., Stearns D. G., Kearney P. A. Investigation of the amorphous-to-crystalline transition in Mo/Si multilayers. J. Appl. Phys. 90, 1017 (2001).

[b25] Bajt S., Chapman H. N., Aquila A., & Gullikson E. M. High-efficiency gratings with asymmetric-cut multilayers. JOSA A 20, 216–230 (2012). 22472750 10.1364/JOSAA.29.000216

[b26] Ersoy O. K. Fourier Optics and Imaging , John Wiley & Sons, Inc.: Hoboken, NJ. USA, (2007)).

[b27] Liao K., Hong Y., Wang Q., Chang G. & Sheng W. Analysis of tilted multilayer Laue lens with stochastic layer thickness error. Opt. Commun. 325, 111–115 (2014).

[b28] Gaudin J. *et al.*. Investigating the interaction of x-ray free electron laser radiation with grating structure. Opt. Lett. 37, 3033–3035 (2012). 22859076 10.1364/OL.37.003033

[b29] Hoekstra H. J. W. M. On beam propagation methods for modelling in integrated optics. Optical and Quantum Electronics 29, 157–171 (1997).

[b30] Yan H. *et al.* Multilayer Laue Lens: A Path Toward One Nanometer X-Ray Focusing. X-Ray Opt. Instrum. 10, 401854 (2010).

[b31] Pennicard D. *et al.* The LAMBDA photon-counting pixel detector. Journal of Physics: Conference Series 425 062010 (2013)

[b32] Thibault P., Dierolf M., Menzel A., Bunk O., David C. & Pfeiffer F. High resolution scanning x-ray diffraction microscopy. Science 321, 379–382 (2008). 18635796 10.1126/science.1158573

