# Supplementary material for: High numerical aperture multilayer Laue lenses
Source: Sci Rep. 2015 Jun 1;5:9892. doi: 10.1038/srep09892 (PMC4450759; doi:10.1038/srep09892)
Supplement: Supporting Information [file srep09892-s1.pdf]

High numerical aperture multilayer Laue lenses

Andrew J. Morgan[1], Mauro Prasciolu[2], Andrzej Andrejczuk[3], Jacek Krzywinski[4], Alke Meents[2], David Pennicard[2], Heinz Graafsma[2], Anton Barty[1], Richard J. Bean[1], Miriam Barthelmess[1], Dominik Oberthuer[1 5], Oleksandr Yefanov[1], Andrew Aquila[6], Henry N. Chapman[1 5 7], and Saša Bajt[2,*]

[1]Center for Free-Electron Laser Science, DESY, Notkestrasse 85, 22607 Hamburg, Germany
[2]Photon Science, DESY, Notkestrasse 85, 22607 Hamburg, Germany
[3]Faculty of Physics, University of Bialystok, K. Ciolkowskiego 1L, 15-245, Bialystok, Poland
[4]SLAC, 2575 Sand Hill Rd., Menlo Park, CA 94025, USA
[5]Dept. of Physics, University of Hamburg, Luruper Chaussee 149, 22607 Hamburg, Germany
[6]European XFEL GmbH, Albert Einstein Ring 19, 22761 Hamburg, Germany
[7]Centre for Ultrafast Imaging, Luruper Chaussee 149, 22607 Hamburg, Germany

*Corresponding author: sasa.bajt@desy.de

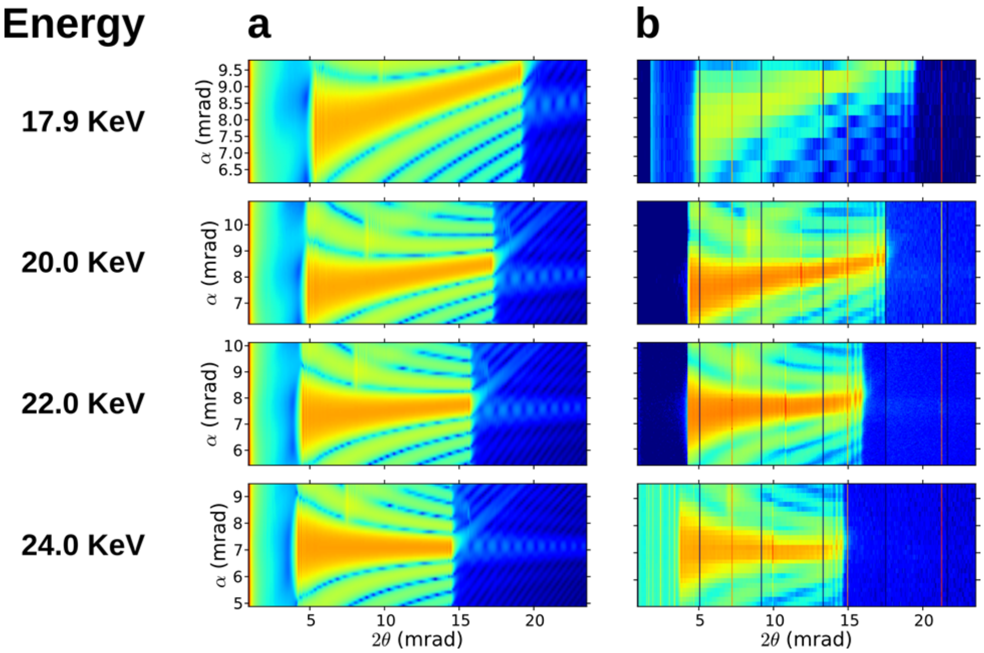

Fig. S1: Diffraction efficiency, as represented by the far-field 1D diffraction of the lens, as a function of tilt (α) of the lens and different photon energies. (a) Simulations for a perfect MLL with wedged layers. (b) Experimentally measured diffraction from our wedged MLL. The stage used to tilt the lens was not encoded and so the tilts are only approximate.
